# Linking Sleep and Racial Health Disparities: Characterizing Sleep in the National Sleep Research Resource

**DOI:** 10.1093/geroni/igab046.2389

**Published:** 2021-12-17

**Authors:** Alycia Sullivan Bisson, Susan Redline, Shaun Purcell

**Affiliations:** 1 Brigham and Women's Hospital, Boston, Massachusetts, United States; 2 Harvard Medical School, Boston, Massachusetts, United States

## Abstract

To address the problem of racial health disparities, prior work has studied differences in environmentally-influenced and modifiable health behaviors, like nutrition and physical activity. Mounting evidence suggests that sleep plays a key role in health, including cardiometabolic and neurodegenerative disease. Thus, studies have begun to characterize sleep differences across racial groups. We aimed to better quantify differences in objective sleep that may contribute to racial health disparities. In preliminary analyses, we examined whole-night polysomnography from 728 individuals between the ages of 7 and 86 (M: 41.39, SD: 19.39) in the diverse Cleveland Family Study (45% males, 57% African Americans; AAs). Linear models examined racial differences in a battery of sleep metrics and tested interactions with age. Microarchitecture metrics included NREM spindle and slow oscillations, important to cognitive-aging and cardiometabolic health. AAs spent relatively more time in lighter N2 (b= 0.295, p<.001) and less time in deeper N3 (b= -0.364, p<.001) sleep. AAs also had lower NREM spectral power across multiple frequency bands (p<.001), and reductions in spindle characteristics including amplitude (b = -0.537, p<.001) and density (b = -0.341, p<.001). Metrics showed qualitatively different patterns of interaction with age: e.g., racial differences in N3 duration increased with age, and differences in spindle amplitude decreased with age (interactions p<.001), despite marked age-related reductions across all individuals. This work may help to identify specific modifiable aspects of sleep as targets for ameliorating health disparities. Patterns of racial differences over the lifecourse may illuminate different mechanisms being active at different points in development.

